# An improved method to detect correct protein folds using partial clustering

**DOI:** 10.1186/1471-2105-14-11

**Published:** 2013-01-16

**Authors:** Jianjun Zhou, David S Wishart

**Affiliations:** 1 JHK Co., Ltd., 2049 Heping Road, Shenzhen, Guangdong, 518010, China; 2 Departments of Computing Science and Biological Sciences, 2–21 Athabasca Hall, University of Alberta, Edmonton, Alberta, T6G 2E8, Canada

## Abstract

**Background:**

Structure-based clustering is commonly used to identify correct protein folds among candidate folds (also called *decoys*) generated by protein structure prediction programs. However, traditional clustering methods exhibit a poor runtime performance on large decoy sets. We hypothesized that a more efficient “partial“ clustering approach in combination with an improved scoring scheme could significantly improve both the speed and performance of existing candidate selection methods.

**Results:**

We propose a new scheme that performs rapid but incomplete clustering on protein decoys. Our method detects structurally similar decoys (measured using either C_α_ RMSD or GDT-TS score) and extracts representatives from them without assigning every decoy to a cluster. We integrated our new clustering strategy with several different scoring functions to assess both the performance and speed in identifying correct or near-correct folds. Experimental results on 35 Rosetta decoy sets and 40 I-TASSER decoy sets show that our method can improve the correct fold detection rate as assessed by two different quality criteria. This improvement is significantly better than two recently published clustering methods, Durandal and Calibur-lite. Speed and efficiency testing shows that our method can handle much larger decoy sets and is up to 22 times faster than Durandal and Calibur-lite.

**Conclusions:**

The new method, named HS-Forest, avoids the computationally expensive task of clustering every decoy, yet still allows superior correct-fold selection. Its improved speed, efficiency and decoy-selection performance should enable structure prediction researchers to work with larger decoy sets and significantly improve their *ab initio* structure prediction performance.

## Background

Predicting the 3D structure of a protein from its sequence continues to be one of the most challenging, unsolved problems in biology. However, thanks to the development of programs such as Rosetta [1] and I-TASSER [2], along with the exponential growth in computational power, it is now possible to predict the structures of small proteins with a high degree of accuracy [2,3]. To be maximally effective, most modern *ab initio* structure prediction programs must generate tens of thousands of candidate structures (called decoys) and then use specially developed heuristic “energy” functions or structure clustering techniques to evaluate and select the top scoring candidates. As a general rule, the performance of an *ab initio* structure prediction program critically depends on: 1) the number of candidate structures generated; 2) the variety of candidate structures generated; 3) the quality of candidate structures generated and 4) the performance of the scoring function and candidate selection criteria. Our focus here is on improving the performance of the latter.

Many studies [4-6] indicate that structural clustering can improve the selection of correct or near-correct folds over a simple heuristic energy function. Shortle *et al.*[4] were probably the first to study the performance of candidate fold detection using structural clustering. They hypothesized that when a reasonably good energy function is used or when a reasonably good structure generation tool is available, most candidate structures generated by a protein structure prediction program will tend to cluster near (but not necessarily on) the correct fold. As a result, cluster centers tend to be closer to the correct fold than other decoys within the cluster. Because of the significant performance enhancements seen with structural clustering, some of the most successful *ab initio* structure prediction programs, such as I-TASSER and Rosetta, now incorporate structural clustering as part of their candidate selection process (see [7] for a more complete survey on clustering algorithms).

While clustering can be quite useful in both candidate fold selection and correct fold detection, its speed deteriorates rapidly as the size of the decoy set increases [8]. Many decoy clustering algorithms, such as SPICKER (v1.0 and 2.0, [5]), which is used by I-TASSER, as well as the clustering algorithm implemented in Rosetta, use a similar clustering approach. In particular, they select the decoy with the largest number of neighbors falling within a certain structural similarity threshold that is either given or detected automatically. The top decoy and its neighbors are selected to form the first cluster. Afterwards the structures in the first cluster are removed from the pool of decoys and the process is repeated until a sufficient number of clusters are identified. Other clustering strategies use hierarchical clustering [9,10] or a travelling salesman approach [11] to aid in structure clustering and candidate selection. All of the above-mentioned strategies require repeated pairwise structure comparisons*.* Therefore, if the size of a decoy set increases 10 fold, then the number of required structure comparisons increases roughly 100 fold. Such a trend can lead to prohibitively poor runtime performance – especially with large decoy sets.

To accelerate the clustering and selection steps on large decoy sets, several methods have been developed in recent years. These include SCUD [12], Calibur [8] and Durandal [6]. SCUD [12] accelerates the clustering process by speeding up the underlying structure comparisons. Rather than performing a time-consuming superposition operation in every pair-wise structure comparison, as is done in traditional decoy clustering algorithms, SCUD superposes every decoy to a reference structure. As a result, SCUD uses the superposition to the reference structure when comparing two structures. The difference between structures, which is called the rRMSD, is essentially an upper bound for the true RMSD (Root-Mean-Square Distance after optimal superposition). Results showed that while the quality of structures selected by SCUD did not improve, the clustering speed improved by a factor of 9 on a decoy set of 2,000 structures.

In contrast to SCUD, Calibur [8] accelerates clustering without changing the structure comparison method. Instead it uses a variety of filtering strategies to limit the number of pairwise comparisons. In the first step, Calibur removes structural outliers that are not similar to a set of randomly sampled decoys by a certain distance threshold. Then, lower and upper bounds derived from the alpha carbon (C_α_) RMSD properties and the triangle inequalities in metric distance are used to reduce the number of structure comparisons. Extensive testing has shown that Calibur is faster than both the Rosetta clustering program and SPICKER (used in I-TASSER). This is especially true when the decoy set is larger than 4,000 structures. The quality of the candidates selected by Calibur has been found to be similar to that of SPICKER.

Durandal [6] combines the lower and upper bounding technique used in SCUD and Calibur with an information entropy technique. For a decoy set of size *n,* Durandal generates a pair-wise distance matrix of size n*(*n-*1)/2 and measures the information entropy by counting the number of “undecided” distances in the matrix. A distance in the matrix is “decided“ if it is clearly below or above a given threshold. Once the matrix is set up, Durandal randomly chooses a set of decoys, computes distances between the decoys, and uses the lower and upper bounds to determine the “undecided” distances so that the entropy in the distance matrix decreases. The algorithm keeps using the lower and upper bounds to decide distances as long as it reduces the entropy in the matrix more efficiently than computing distances. Tests show that Durandal is faster than both SCUD and Calibur, by a factor of up to 3.4 on a decoy set of 21,080 structures. Not only is Durandal faster, it was also shown to improve upon the quality of folds that could be selected. In particular, on 40 large I-TASSER decoy sets, Durandal selected a more correct fold than the I-TASSER energy function (alone) in 25 out of 40 cases versus just 20 for SPICKER. However, as noted by its authors, Durandal is not very efficient with memory usage due to the quadratic size of the distance matrix [6]. A recently updated version of Durandal applies a Quaternion-based Characteristic Polynomial method [13] to accelerate its RMSD calculations. This change has improved Durandal’s overall runtime by up to 25%.

With continuous algorithmic improvements over the last decade, many *ab initio* structure prediction methods are now running significantly faster than earlier methods. For example, I-TASSER was reported to take just 5 hours to model the structures of certain smaller proteins on a single CPU [2]. Presumably this involved the generation of thousands of decoy structures. Running multiple copies of I-TASSER on multiple CPUs (or a faster 2012 vintage CPU) would no doubt reduce the sampling time significantly (i.e. minutes). However, clustering large decoy sets with existing methods such as Durandal can still take at least half an hour -- as shown in later in this paper. Furthermore, to the best of our knowledge, currently there is no parallelized version of Durandal (or any other structure clustering program) available. This suggests that structural clustering is becoming a significant time bottleneck in *ab initio* structure prediction. Clearly the improvement of clustering speed would benefit a number of state-of-the-art structure prediction methods such as I-TASSER.

In this work we describe a faster, more efficient and more accurate approach to detect correct protein folds using a technique called partial clustering. Given that the ultimate goal of clustering in decoy selection is to return representatives of the decoy clusters, rather the clusters themselves, then we would argue that partial clustering should be sufficient to solve the problem at hand. In particular, we have designed a partial clustering algorithm that does not need to define cluster membership for every decoy but is able to extract key representatives quickly. Our method also combines the speed of this fast clustering approach with a more intelligent approach to scoring or ranking candidate folds. In contrast to other quality assessment methods, our method uses the scoring energy to rank a small set of cluster centers instead of the whole decoy set. We have found that our clustering strategy can be applied to any scoring function to enhance the rate of correct fold detection. Tests conducted on decoy sets generated by Rosetta and I-TASSER show that our method is able to select a higher proportion of correct folds than using energy functions alone or using other fast (or traditional) structure clustering algorithms. Speed and efficiency testing also shows that our method is up to 22 times faster than Durandal (the fastest clustering method described to date), and that it is also significantly more memory-efficient.

## Methods

Our method, called HS-Forest, is based on the concept of partial clustering and then “intelligently” combining this partial clustering with energy function evaluation to detect the most correct fold from a given decoy set. The first step in the program involves using a novel structural clustering scheme to detect structurally “dense” regions in the given decoy set. These regions are thought to be local minima in the protein folding energy landscape according to the hypothesis that most candidate structures generated by a protein structure prediction program will tend to cluster near the correct fold if the structure generation program is reasonably good [4]. In the clustering stage, we extract a representative for each cluster without assigning every object to a cluster. We then compute the distances between the extracted representatives and a small number of lowest energy decoys. Unless otherwise specified, in this paper we measure the distance between two decoys using the C_α_ RMSD distance (the RMSD calculated using only C_α_ atoms). However, it is important to note that our method is applicable to both metric and non-metric distance functions. Once this extracted representative distance calculation is done, we then select a representative with the smallest total distance to the lowest energy decoys as a candidate for the best decoy. In the clustering stage we introduce a random factor, described later, so that each clustering process will typically generate a different optimal candidate. We run the clustering process multiple times to generate a set of candidates. From this set of candidates, we return a consensus decoy that has the smallest total distance to all other candidates. The algorithm is outlined step-by-step below:

1. Select a given number of pivots randomly;

2. For each pivot create a hashing function;

3. Build the root node of a tree, which is the first leaf of that tree;

4. Randomly select a hashing function to split the leaves of the tree;

5. Go to Step 4 until the tree reaches a certain height as described below;

6. Determine the cluster nodes;

7. For each of the largest cluster nodes, select a representative;

8. Rank the representatives by their total distances to the top energy decoys. The top one is the candidate of the tree;

9. Go to Step 3 until a given number of trees are constructed;

10. Return the consensus of the candidates as the best decoy.

To efficiently detect locally “dense” regions in a given decoy set we decided to use a recently developed database searching technique called *Local Sensitive Hashing*[14], or *LSH.* The idea of LSH is to design a set of hashing functions so that similar objects have a probability (although not necessarily a high probability) of being hashed to the same bin by each hashing function. Even though every individual hashing function is not perfect and can hash dissimilar objects to the same bin, by combining multiple hashing functions we can still manage to group similar objects together.

Our design of the hashing function is based on a well-known metric property [15-17] that two similar decoys usually have similar distances (as measured by RMSD) to a third decoy. First, our algorithm randomly selects a small set of decoys from the given decoy set as *pivots* and computes the distances between these pivots and all decoys in the decoy set. Then, for each pivot our algorithm divides all the decoys into two bins, depending on whether the distance between the pivot and the decoy is less than the median of all distances to the pivot. Similar decoys are likely to have similar distances to the pivot and thus have a higher probability to be placed in the same bin as opposed to different bins. From each pivot, our algorithm constructs a hashing function. We use the median distance to divide the decoys because it makes the bin size of the hashing function more balanced.

To organize the hashing functions, our algorithm constructs a tree, denoted as an *HS-Tree*. “HS” in HS-Tree and HS-Forest stands for “Height and Size”. These two letters come from the two factors behind our clustering algorithm, both of which are used to define the clusters. Starting from the whole decoy set, our algorithm splits the decoys into two nodes by applying a randomly chosen hashing function, with each bin of the hashing function corresponding to a node. Each of the two nodes is further split by another randomly chosen hashing function, i.e., each layer of the tree is split by a randomly chosen hashing function. This process is repeated until a certain height to the tree is reached. At the beginning of the process, the nodes in the tree can contain very dissimilar decoys, but as the nodes are split further down the tree, dissimilar decoys are more likely to be separated into different nodes while similar decoys are more likely to remain together thereby representing a “denser” region.

The next step is to decide which nodes contain a dense region, otherwise known as a cluster. Ideally, a cluster is a region balanced with density and size (i.e., number of decoys). Since the density of a node is indicated by its height in an HS-Tree, the ideal clusters are in nodes balanced by both height and size. We define such a node as a *cluster node* as given below.

DEFINITION 1 (Cluster node). In an HS-Tree *t* of height *H* (≥ 1) and size *Z* (≥ 2), let *p* be a path in *t* from the root to a leaf node, a cluster node in *p* is a node *v* of height *h*(*v*) and size *z*(*v*) so that

(1) $$\left\{ \begin{array}{c} \frac{h\left(v\right)}{H}\geq \frac{\log z\left(v\right)}{\log Z}\\ v=\underset{v\in p}{\arg\;\min}h\left(v\right) \end{array}\right.$$

**Figure 1:**
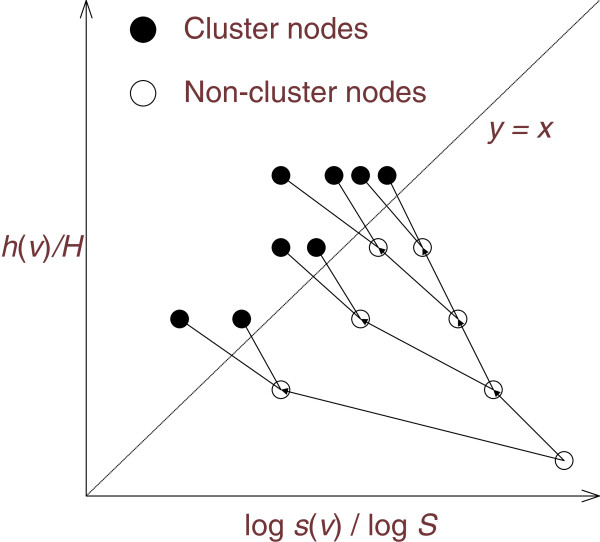
**An example of an HS-Tree.** Cluster nodes are nodes balanced with density (height) and size. Sub-trees under each cluster nodes are omitted.

Intuitively, as shown in Figure 1, a cluster node is a node near the line *y=x* in a 2D plot with *y*= log(*z*(*v*))/log(*Z*), and *x=h*(*v*)*/H.* We apply a log scaling function to normalize the node size since the node size in an HS-Tree usually decreases in an exponential manner when traveling from the root to the leaves. Each path in an HS-Tree from the root to a leaf can contain at most one cluster node. Note that in our algorithm, not every decoy is necessarily assigned to a cluster node. On the other hand, each HS-Tree must contain at least one cluster node, as guaranteed by the lemma below.

LEMMA 1. In an HS-Tree *t* of height *H* (≥ 1) and size *Z* (≥ 2), let *c* be the number of cluster nodes in *t*, then *c* ≥ 1.

*Proof synopsis*: Since the first hashing function splits the decoy set in half by the median distance with each half close to *Z/*2 and no larger than Z*2/3 (equal only when *Z* = 3), for any node of height *h*(*v*)=*H* and size *z*, log(*z*)/log(*Z*) ≤ log(Z*2/3)/log(*Z*). Since log(Z*2/3)/log(*Z*) = 1-log(3/2)/log(*Z*), log(*z*)/log(*Z*) < 1–0.40546/log(*S*) < 1. Given that *h*(*v*)/*H* = *H/H =* 1, log(*z*)/log(*Z*) <*h*(*v*)/*H*, and equation (1) is satisfied. Therefore, *c* ≠ 0.

Having extracted the clusters from the non-redundant set of cluster nodes, the next step is to extract a representative from each cluster. At this stage, the challenge is two-fold: (1) even though each cluster is likely to consist of decoys structurally similar to each other, it is possible that some dissimilar decoys are mixed into the cluster; and (2) the size of clusters can vary significantly so that the traditional approach of computing a medoid with the minimal total distance to the decoys within a cluster can involve a quadratic number of distance computations. In our algorithm, we select a representative of a cluster by choosing the decoy with the smallest total distance to the set of pivots we picked when constructing the hashing functions. No additional distance computation is needed.

To select the best decoy from the pool of cluster representatives, we borrow an idea used by [18], which ranks every decoy in a decoy set by its total distance to the lowest energy decoys. In our algorithm, for given numbers *S* (the number of clusters to consider) and *E* (the number of lowest energy decoys), we select the *S* largest clusters and rank their representatives by the total distance to the *E* lowest energy decoys (the values of *S* and *E* are further elaborated upon in the Discussion section). The additional distance computation is minimal when *S* and *E* are small. The highest ranked representative is a candidate for the best decoy in the decoy set.

Due to the random factor we introduced when constructing our HS-Tree, different HS-Trees can return different candidates for the best decoy. To compute a consensus, our algorithm generates multiple HS-Trees. First a set of *P* pivots are randomly selected and *P* corresponding hashing functions are created. Then, *H*_*max*_ hashing functions are randomly chosen to build an HS-Tree, so that the maximal height of the tree is *H*_*max*_. This process is repeated until a given number (typically between 5 and 50) of HS-Trees are constructed. Finally, our algorithm ranks the candidates from all the trees by the total distance to all the candidates, and returns the highest ranked candidate.

In our implementation, when constructing HS-Trees and extracting cluster nodes, we use *H*_*max*_ to replace the actual tree height *H*. In large decoy sets, these two values are usually the same. The advantage of using this approximation is that we can construct only a portion of the whole tree and stop splitting whenever the inequality (1) in Definition 1 is satisfied. This significantly reduces the runtime. The disadvantage is that, theoretically, we can no longer guarantee that the returned result is not empty. However, this appears to be a rare event as an empty result was never returned in our experiments with 75 different decoy sets consisting of between 1,135 to 64,307 structures. In the worst case, if an empty result is returned, the user can simply reduce *H*_*max*_ and re-run the program.

## Results

We tested our method, *HS-Forest*, on a large collection of Rosetta and I-TASSER decoys. The Rosetta decoys consist of 35 decoy sets for different protein targets with a diverse size range from 1,135 to 64,307 structures. The I-TASSER decoys consist of 40 large decoy sets [2] with varied size between 12,499 and 32,000 structures, and were used to test Durandal [6]. Both the Rosetta and I-TASSER decoys were generated by a combination of *ab initio* and fragment assembly methods, and each decoy set contained at least one decoy having a C_α_ RMSD below 4 Angstroms relative to the native (i.e. correct) structure. The exact sizes of the Rosetta and I-TASSER decoy sets are shown in Additional files 1 and 2, respectively.

### Speed and efficiency

To evaluate the speed and efficiency of HS-Forest, we tested it on both the Rosetta and I-TASSER decoy sets. Given that the results presented by [6] clearly showed that Durandal was faster than SCUD and Calibur, in our experiments we only compared HS-Forest with Durandal (without Quaternion-based Characteristic Polynomial methods to accelerate RMSD calculations [13]) and Calibur-lite [8]. Calibur-lite is a simpler, faster program than Calibur that only outputs the best decoy. For our tests, Durandal was also set to output the best cluster only, using a value of 0.05 for the semiautomatic threshold detection as suggested by its authors [6]. All reported runtime values are measured by the Linux program “time” and averaged over 10 runs on a dual Quad-Core AMD Opteron 2378 Linux sever with 16 GB of RAM using only a single core.

**Figure 2:**
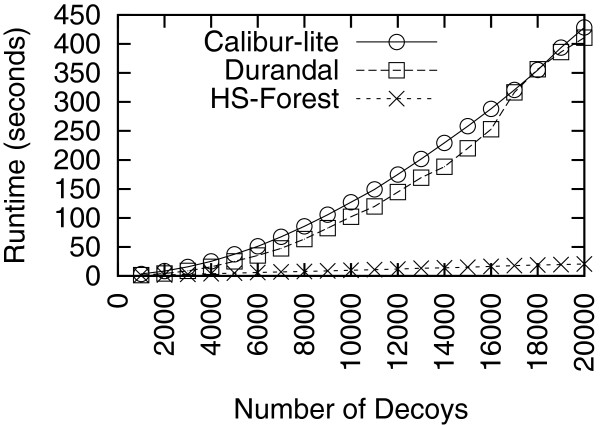
**Runtime on the 1shfA decoy set.** The size varies from 1,000 to 20,000 structures.

The first test set we investigated was the 1shfA set from the I-TASSER decoys. We varied the decoy set size from 1,000 to 20,000 by randomly sampling a portion of the entire decoy set. The runtimes for the three methods are shown in Figure 2. As seen in this figure, HS-Forest when using the I-TASSER energy function significantly outperforms both Durandal and Calibur-lite. Furthermore the speed gap increases as the size of the testing set increases. Using the full set of 20,000 decoys, HS-Forest achieves a speedup of 20 over both Durandal and Calibur-lite.

**Figure 3:**
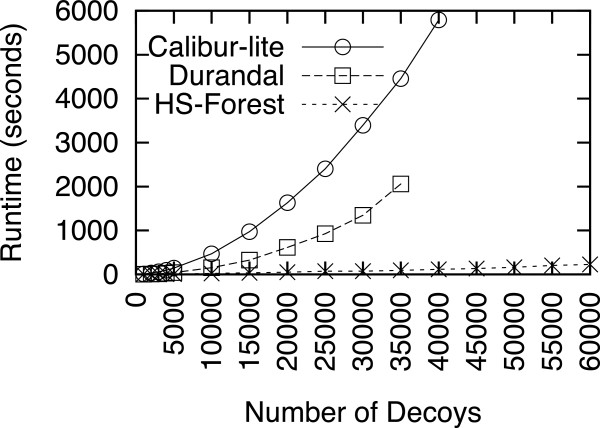
**Runtime on the 1bm8 Rosetta decoy set.** The size varies from 1,000 to 60,000 structures. Durandal runs out of memory when the size exceeds 40,000 proteins. Calibur-lite takes more than 2 hours to finish a run when the number of proteins was more than 45,000.

The second test set we studied was the 1bm8 set from the Rosetta decoys. This decoy set contains 64,307 decoys and poses a significant challenge to most clustering algorithms. Similar to the first experiment, we varied the decoy set size from 1,000 to 60,000. The runtime for all three methods are shown in Figure 3. Durandal runs out of memory once the number of decoys exceeds 35,000, while Calibur-lite takes more than 2 hours to finish a run as soon as the size exceeds 45,000 decoys. HS-Forest using the Rosetta energy function finishes all calculations and outperforms Durandal and Calibur-lite consistently, requiring just 228 seconds to analyze 60,000 decoys. The speed-up factors for HS-Forest over Durandal and Calibur-lite are 22 and 49 respectively.

Figures 2 and 3 also show that the performance of a given clustering method can be quite different for different decoy sets. For instance, we can see that Calibur-lite has a much slower runtime than Durandal in Figure 3 but its runtime is about the same as Durandal in Figure 2. To compare the performance on diverse decoy sets of varying size, we compared the runtime for all three methods on an independent collection of 34 Rosetta decoy sets (excluding 1bm8). These decoy sets are smaller than the 1shfA and 1bm8 sets, but have a size range from 1,135 to 17,934 decoys. HS-Forest had the shortest runtime among the three methods for all 34 sets, achieving a speedup factor of 1.4 to 14.0 over Durandal, and 2.1 to 10.2 over Calibur-lite. The detailed results can be found in Additional file 1.

HS-Forest is also very memory efficient. While Durandal ran out of memory when testing the 1bm8 set from the Rosetta decoy set on our testing machine with 16 GB RAM, HS-Forest was able to finish analyzing all the decoys in this set with the maximal memory usage of around 3 GB.

The computational time complexity for HS-Forest can be calculated as follows: let the number of decoys be *N*, then the computational time complexity to compute the *P* hashing tables is *O*(*PN*log*N*). The complexity to construct an HS-Tree is *O*(*N*log*N*). Therefore the total time complexity of HS-Forest is *O*((*P*+*T*)*N*log*N*) where *T* is the number of trees.

### Performance for correct fold detection

In this section, we will show that HS-Forest is able to improve the performance of energy functions in correct fold detection. We will also compare HS-Forest with two state-of-the-art decoy clustering programs: Durandal and Calibur-lite. Since both Rosetta and I-TASSER have their own clustering programs and since these clustering programs might have an advantage when tested on their own decoy sets, we also included these two clustering programs in our comparison. The results show that HS-Forest exhibits better performance than all the above-mentioned methods.

Measuring the performance for correct fold detection is a challenging task because the same method usually performs differently on different decoy sets. For example, a method can outperform a standard energy function significantly in one decoy set but consistently fail to outperform the same energy function in other decoy sets. To measure performance we adopted two different criteria. For the first criterion, denoted as *Criterion-1*, the percentage of decoys that the top-scoring decoy outperformed is averaged over all decoy sets is used as the metric. Criterion-1 measures, on average, how close the selected decoy is to the native (i.e. correct) structure compared with the other decoys in the decoy set. The second criterion, denoted as *Criterion-2*, was adopted from [6]. It essentially measures how frequently a given method can outperform the energy function in different decoy sets. Specifically it measures the frequency with which the top decoy selected by a given method has a lower C_α_ RMSD than the top decoy selected by the energy function alone. Since both HS-Forest and Durandal contain random seeding functions, unless otherwise specified, we averaged their results over 50 runs.

For our experiments on the Rosetta decoys, we applied HS-Forest to enhance the decoy selection using different energy functions, including the Rosetta energy values (that came with the decoys), the GaFolder pseudo-energy values [19], and a consensus score (denoted as Consensus) that ranks decoys by the total C_α_ RMSD distance to a number of reference structures. To calculate the Consensus score, we chose the same number of reference structures as the number of pivots used in HS-Forest. When HS-Forest was combined with an energy function, we use that function to select the 10 lowest energy decoys in HS-Forest. The results are shown in Table 1. Under the Criterion-1 column, we can see that HS-Forest is able to enhance the decoy selection performance for all three energy functions with a percentage difference of 5.2% (or a percentage ratio improvement of 6.5%) for the Rosetta energy, and a percentage difference of 8.6% (or a percentage ratio improvement of 12.0%) for the GaFolder energy. Since HS-Forest already used the Consensus method to select a representative for each cluster, we would expect the HS-Forest results would be similar to that of the Consensus method alone. Nonetheless, HS-Forest still outperformed the Consensus result by a percentage difference of 2.1% (or a percentage ratio of 2.6%). Under the Criterion-2 column, we compare how frequently different methods can perform against the baseline Rosetta energy function. On average, when the Rosetta energy is used in HS-Forest it outperforms the Rosetta energy, alone, in 24.7 out of the 35 decoy sets (71%). The GaFolder and Consensus energy functions outperformed the Rosetta energy in just 15 and 17 out of 35 cases respectively. However, after applying the HS-Forest algorithm to the same energy functions, the results improved to 20 and 18 out of 35 cases, respectively.

**Table 1:** Performance on Rosetta decoys

**Energy/Program**	**Method**	**Criterion-1 (%)**	**Criterion-2 (/35) ± Std Dev.**
Rosetta	Energy only	80.0	NA (baseline)
	with HS-Forest	85.2	24.7 ± 1.8
GaFolder	Energy only	71.8	15.0
	with HS-Forest	80.4	20.0 ± 1.2
Consensus	Energy only	81.2	17.0
	with HS-Forest	83.3	18.0 ± 0.7
Durandal		82.5	16.0 ± 1.8
Calibur-lite		78.9	13.6 ± 0.7
Rosetta	Clustering	80.0	20

Criterion-1 and Criterion-2 are defined in the manuscript. Criterion-1 measures the percentage of decoys that the top-scoring decoy outperformed; Criterion-2 measures the frequency with which the top decoy, selected by a given method, has a lower C_α_ RMSD than the one selected by the energy alone. The values behind ± are the standard deviations among different runs.

On the Rosetta decoys, we also compared HS-Forest with Durandal, Calibur-lite, and the clustering program used in the recently released Rosetta 3.4. When testing on the largest decoy set, 1bm8, Durandal ran out of memory and Calibur-lite took more than 2 hours to finish. Consequently, we used the largest sample that Durandal and Calibur-lite could generate on the 1bm8 set to calculate its performance for this particular decoy set (since Calibur-lite was much slower than other methods on large decoy sets, we only repeated its run for a total of 10 times). The average result for Durandal using Criterion-1 is 82.5%, which is better than all of the energy-only methods but is still 2.7% less than what Rosetta energy function combined with HS-Forest achieved. For Criterion-2, the average result for Durandal was 16.0 out of 35 decoy sets (46%) versus 24.7 (71%) for Rosetta with HS-Forest. This result for Durandal is somewhat worse than what HS-Forest achieved even when it used the GaFolder and Consensus energy functions. Similar to Durandal, the performance of Calibur-lite and the Rosetta clustering program in Table 1 are worse than what was achieved when the Rosetta energy function was combined with HS-Forest.

The results on the I-TASSER decoy sets are shown in Table 2. The energy functions we tested include the I-TASSER values that come with the decoys, the GaFolder energy and the Consensus score. Using Criterion-1, similar to the results on the Rosetta decoys, HS-Forest improves the decoy selection for all three energy functions. Under Criterion-2, we used the I-TASSER energy alone as the baseline method. As seen in Table 2, HS-Forest methods consistently outperform the energy-only methods, with an improvement of 8.3 cases for GaFolder and 4.5 cases for the Consensus method. On this data set we also compared HS-Forest with Durandal, Calibur-lite, and SPICKER 2.0 (the clustering program in I-TASSER). The performance of Durandal for Criterion-1 and Criterion-2 is 72.9% and 25.7/40 respectively, which is worse than what was obtained using GaFolder with HS-Forest (75.3% and 27.3/40). The performance of Calibur-lite for Criterion-1 and Criterion-2 is 74.1% and 26.6/40 respectively, which is slightly worse than those of GaFolder with HS-Forest. For SPICKER 2.0, we used the clustering results downloaded from its website to calculate the scores. More specifically, we used the so-called “Closc” decoys that come with the decoy sets to calculate the Criterion-1 and Criterion-2 scores. A “Closc” decoy is the decoy closest to the first SPICKER cluster centroid. It has been shown that these “Closc” decoys have a lower RMSD to the native structure than the SPICKER average structure after removing most steric clashes [20]. The results shown in Table 2 also indicate that SPICKER 2.0 exhibits a poorer performance than GaFolder with HS-Forest using both Criteria.

**Table 2:** Performance on I-TASSER decoys

**Energy/Program**	**Method**	**Criterion-1 (%)**	**Criterion-2 (/40) ± Std Dev.**
I-TASSER	Energy only	59.7	NA (baseline)
	with HS-Forest	68.4	27.2 ± 1.9
GaFolder	Energy only	57.1	19
	with HS-Forest	75.3	27.3 ± 1.8
Consensus	Energy only	67.4	22
	with HS-Forest	72.1	26.5 ± 1.2
Durandal		72.0	25.7 ± 1.2
Calibur-lite		74.1	26.5 ± 0.5
SPICKER 2.0		72.6	25.0

Criterion-1 measures the percentage of decoys that the top scoring decoy outperformed; Criterion-2 measures the frequency with which the top decoy selected by a given method has a lower C_α_ RMSD than the one selected based on energy alone. The values behind ± are the standard deviations among different runs.

Because the performance of HS-Forest on the I-TASSER data appears to be modestly better than the performance of Durandal and Calibur-lite, we performed an unpaired Student’s t-test to assess the statistical significance [21] of this performance difference. The choice of a Student’s t-test was based on the fact that the Criterion-1 and Criterion-2 scores exhibited a normal (i.e. Gaussian) distribution over the 50 sample runs performed with each program (due to its longer runtime, Calibur-lite was run 10 times and compared with 10 random HS-Forest runs) and the fact we were interested in determining whether the average Criterion scores were statistically different. For Durandal the t-test p-values are 6.1x10^-13^ and 6.1x10^-7^ for Criterion-1 and Criterion-2 respectively; for Calibur-lite the p-values are 3.4x10^-4^ and 6.3x10^-3^ for Criterion-1 and Criterion-2 respectively. So using the standard p<0.05 criterion for statistical significance these differences are statistically significant. We also calculated the standard deviation on the C_α_ RMSD values, relative to the native structure, among different runs for each decoy set. These are found in Additional files 1 and 2. The average standard deviation is 0.28 and 0.13 Angstroms for the Rosetta and I-TASSER data sets, respectively.

We also tested HS-Forest using another distance metric known as the GDT-TS distance [22,23] as implemented in TMscore [24]. Since there is no trivial way to run Durandal and Calibur-lite using a distance metric other than C_α_ RMSD, we calculated the GDT-TS scores using TMscore for the top decoys selected by Durandal and Calibur-lite. The following results were averaged over 10 runs. On the Rosetta decoys, the average performance of Rosetta energy with HS-Forest for Criterion-1 and Criterion-2 was 81.9% and 22.0/35 respectively. These values are better than the performance achieved with Durandal (77.0% and 18.8/35) and Calibur-lite (71.2% and 14.3/35). On the I-TASSER decoys, the average performance using the GaFolder energy with HSForest for Criterion-1 and Criterion-2 were 75.2% and 27.5/40 respectively, which is also better than that found for Durandal (72.9% and 25.7/40) and Calibur-lite (73.9% and 25.9/40).

## Discussion

### Parameter settings

HS-Forest contains several parameters that can be adjusted, including the number of pivots *P*, the maximal tree height *H*_*max*_, the number of trees *T*, the number of largest clusters to consider *S*, and the number of lowest energy decoys *E*. In our experiments, to reduce the number of parameters while at the same time maintaining randomness among the trees, we always set *H*_*max*_ = *P*/2. This is done so that different trees have partially different sets of hashing functions to generate different candidates for the final consensus stage. While increasing *P*, *T, S* or *E* generally increases the runtime, how each affects the program’s performance is not particularly obvious. To study the impact of these parameters, we tried different values for the 4 parameters on the I-TASSER decoys using the I-TASSER energy function with HS-Forest. Unless otherwise specified, for all the experiments we set *P* = 40, *T* = 30, *S* = 30, and *E* = 10. Overall, the results show that the performance of HS-Forest is not particularly sensitive to these parameters.

**Figure 4:**
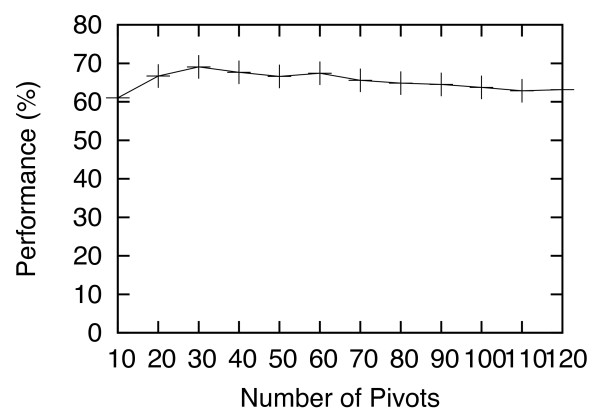
**Criterion-2 performance (in %) of I-TASSER with HS-Forest, for the number of pivots *P *varies from 10 to 120.**

The value of *P* is probably the most important parameter in HS-Forest. Figure 4 shows the Criterion-2 performance (in %) when *P* is varied from 10 to 120. At the lowest *P* of 10, HS-Forest is least accurate. As *P* increases, the performance also increases and reaches a maximal value when *P* = 30. Afterwards, the performance declines slowly. We conjecture that the reason for the slow decline is that when *P* (and *H*_*max*_) values are too large, it makes the inequality (1) in Definition 1 less optimal to extract clusters. Based on these data we recommend a *P* value between 20 and 60.

**Figure 5:**
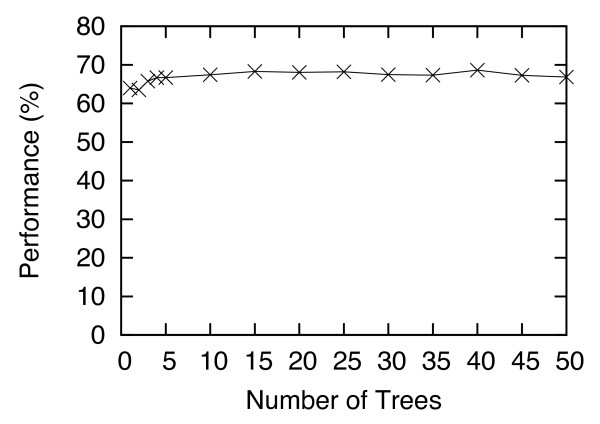
**Criterion-2 performance (in %) of I-TASSER with HS-Forest, for the number of trees *T *varies from 1 to 50.**

**Figure 6:**
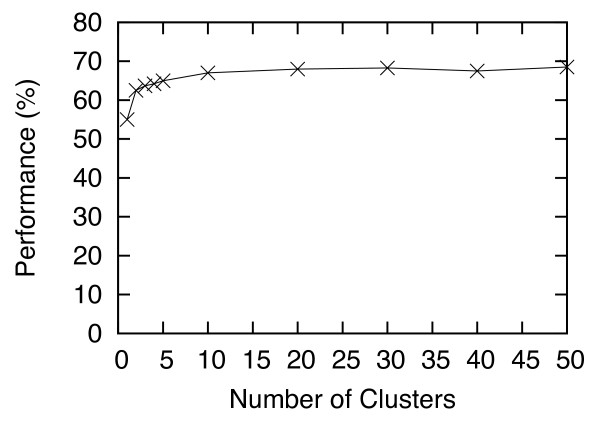
**Criterion-2 performance (in %) of I-TASSER with HS-Forest, for the number of largest clusters *S *varies from 1 to 50.**

**Figure 7:**
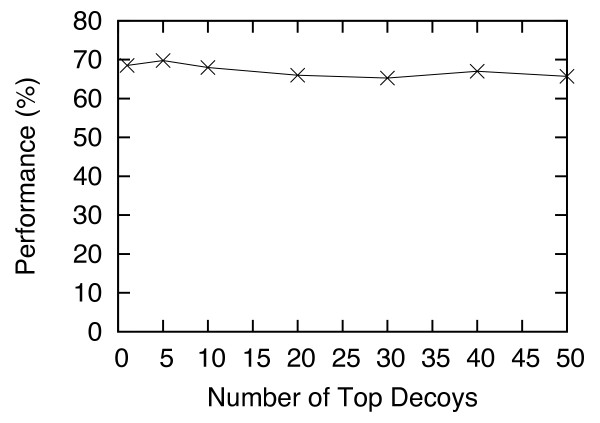
**Criterion-2 performance (in %) of I-TASSER with HS-Forest, for the number of top decoys *E *varies from 1 to 50.**

Figure 5 shows the Criterion-2 performance (in %) with the number of trees (*T*) varied from 1 to 50. This graph shows that when *T* is smaller than 5, the performance is suboptimal, but for *T* ≥ 15, the result is stable. This result also indicates that the final step of HS-Forest, which involves computing the consensus of the results from multiple trees, does improve the performance. In other words, the consensus of multiple trees (> 5) gives a better performance than a single tree. Figures 6 and 7 show how the Criterion-2 performance (in %) varies with respect to the numbers of largest clusters *S* and lowest energy decoys *E*. For *S* > 10, the performance levels off; and *E* does not have a significant impact on the performance.

For all the experiments presented in the Results section, we set *P =* 40, *T =* 30, *S* = 30, and *E =* 10 on both the Rosetta and I-TASSER data. The parameters were selected based on “training” with the I-TASSER data using the C_α_ RMSD distance, and then tested on the leave-out Rosetta data set. As seen in the last section the performance of HS-Forest remained superior even on the leave-out data. To further validate this result we also tested the same parameters on I-TASSER and Rosetta data using the GDT-TS distance, which served as another leave-out testing set. Again, our results show that HS-Forest’s performance was not compromised. The superior performance of HS-Forest on all leave-out sets, as well as the data shown in Figures 4, 5, 6, 7 shows that these parameter settings are quite robust and certainly general enough to be applied on other decoy sets using different similarity metrics.

### The role of clustering

Our method combines the power of partial clustering with the use of energy functions to help detect correct folds. It is interesting to see how clustering contributes to the improved performance of HS-Forest. To explore this further, we tested the Rosetta decoys by simply ranking the decoys by the total C_α_ RMSD distance to the 10 lowest energy decoys. When using the 10 lowest Rosetta energy decoys, the performance is 81.8% for Criterion-1. Compared with the results in Table 1, this Criterion-1 performance is only 1.8% better than what is obtained when using the Rosetta energy only, while it is 3.4% lower than that of Rosetta with HS-Forest. A similar result is obtained when using the 10 lowest GaFolder energy decoys, with a Criterion-1 performance just 1.8% better than that obtained using the GaFolder energy alone and 6.8% lower than that of GaFolder with HS-Forest. These results show that without partial clustering, the idea of using the 10 lowest energy decoys as reference structures to rank decoys is only slightly better than using the energy functions alone.

Essentially, the clustering step in HS-Forest narrows the collection of decoy candidates to smaller set that are close to the cluster centers. As hypothesized by [4], these structures are more likely to be closer to the correct fold. While the energy/consensus ranking in HS-Forest is not perfect, we conjecture that the initial focus on selecting good candidates helps improve the Criterion-1 performance mentioned above. Another factor that may contribute to the performance improvement seen in HS-Forest is the use of a consensus of multiple random trees, as shown in Figure 5.

On the other hand, clustering on its own may also have problems discerning the correct fold in certain situations, particularly when there are multiple clusters of nearly equal size. In these cases, choosing the wrong cluster could lead to a very poor result. This is where other information, such as the energy score ranking, can help. It is for these reasons that HS-Forest combines both structure clustering and energy score information to detect correct folds.

### Improvements to energy functions

**Figure 8:**
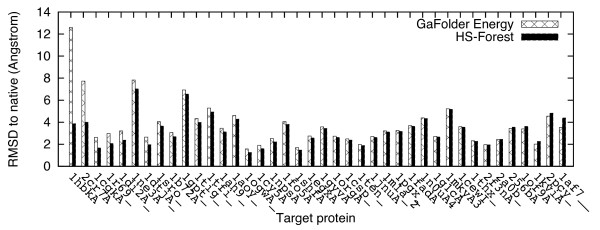
**Comparison of C**_**α **_**RMSD to the native structure for the selected decoys on the I-TASSER decoy sets before and after applying HS-Forest to GaFolder energy, sorted by the C**_**α **_**RMSD difference.** HS-Forest results are averaged over 50 runs.

Our results on the Rosetta and I-TASSER decoys show that HS-Forest is more effective at detecting correct folds for certain energy functions than for others. As seen in Table 1, GaFolder with HS-Forest has the largest performance enhancement over its energy-only method. A similar trend is observed in the I-TASSER results presented in Table 2. When using energy-only methods, the Criterion-1 performance of GaFolder is somewhat worse than those of the I-TASSER energy and Consensus score on the I-TASSER decoys. However, after applying HS-Forest, the Criterion-1 performance of GaFolder is improved by 18.2% and it actually performs the best among all methods. This improvement can be explained by inspecting Figure 8. As seen here, the energy-only method of GaFolder chooses particularly poor quality decoys for two decoy sets: 1hbkA and 2cr7A. These mistakes were corrected after applying HS-Forest so that the C_α_ RMSD to the native structure drops from 12.6 to 3.9 Å and from 7.7 to 4.0 Å respectively.

The significant improvements seen with GaFolder indicates that our HS-Forest concept may open the door for computational biologists to reconsider using some previously discarded heuristic energy functions in structure prediction and refinement. In some cases the inferior overall performance for some energy functions may be due to a very poor performance on a small subset of proteins. Such weaknesses could be corrected by using HS-Forest as part of the energy function or as part of the evaluation criteria.

This result also illustrates the advantage of HS-Forest over traditional clustering algorithms in that it is able to combine different energy functions and adapt to different types of decoy sets. Decoys generated by different structure prediction programs can often have very different properties. Therefore it is important to be able to use different energy functions to detect the most correct folds. While traditional clustering algorithms only rely on structure comparisons within a given decoy set to select correct folds, HS-Forest combines efficient structure clustering with different energy functions to generate better decoy selections.

### Performance on small decoy sets

To test the performance of HS-Forest on small decoy sets, we downloaded just such a set located on the I-TASSER website [25]. This is a non-redundant subset of the original I-TASSER decoys that were structurally refined via GROMACS 4.0. These very small decoy sets vary in size from just 270 to 574 structures. We ran HS-Forest on this set and compared the results with the SPICKER results that come with the data set. In order to be able to compare the scores on the whole I-TASSER data set as well, we used the values of the whole set as a reference when calculating the Criterion-1 and Criterion-2 scores. It turns out that the SPICKER results on this new data set are actually worse than the SPICKER results obtained on the whole set in Table 2, with a Criterion-1 score of 71.9% and a Criterion-2 score of 20/40. This indicates that clustering over a reduced and/or refined subset might not necessarily have an advantage over clustering on the whole decoy set. As might be expected, HS-Forest shows no advantage over SPICKER on these very small decoy sets. This is because HS-Forest is designed to run on much larger decoy sets. Nonetheless, HS-Forest still enhances the performance of the GaFolder energy on this new data set. The Criterion-1 and Criterion-2 scores for HS-Forest using the GaFolder energy are 60.4% and 21.1/40 respectively, which are better than those of using the GaFolder energy only (45.4% and 15/40 respectively). Similar to the case with SPICKER, the scores of HS-Forest and GaFolder energy (alone) are also worse than those of the whole data set.

### Drawbacks of HS-Forest and decoy clustering programs

HS-Forest is not without some limitations. First of all, HS-Forest may not work particularly well with poor quality decoy sets. Following the previous work of Durandal, we limited our testing data to those decoy sets with at least one decoy being less than 4 Å C_α_ RMSD away from the native structure. To see how well HS-Forest would perform on decoy sets that do not satisfy this condition, we tested HS-Forest on 16 I-TASSER decoy sets from [2]. These decoy sets were left out from our experiments in the Results section because they do not satisfy the above-mentioned RMSD condition. The Criterion-1 score of HS-Forest with the GaFolder energy on this data set is 56.3%, which is somewhat higher than those of GaFolder energy alone (47.9%) and I-TASSER energy alone (51.8%). For all the three methods, their scores were found to be much worse than the results in Table 2 for the 40 I-TASSER decoy sets. The significantly lower Criterion-1 performance for all three methods on the 16 poor quality decoy sets indicates that not just the poor quality sets will have overall poorer quality decoys, but the decoy selection methods are also less effective on such decoy sets (the Criterion-1 score indicates how high the return structure is ranked within the decoy set), making it harder to return useful models from these decoy sets than from good quality decoy sets.

A second shortcoming with HS-Forest is that the use of a random factor leads to a non-deterministic output. The speed gains with HS-Forest rely primarily on randomized hashing and the fact that it does not perform a full clustering. These changes make its clustering results a little less stable than other clustering methods. To reduce this effect, HS-Forest creates multiple trees and computes the consensus. As we can see in Tables 1 and 2, its standard deviations on Criterion-2 score are close to those of Durandal.

## Conclusion

In this work, we have proposed a novel partial clustering scheme for decoy selection in protein structure prediction. This method, called HS-Forest, avoids the computationally expensive task of clustering every decoy, yet still allows superior correct-fold selection. The basic idea behind HS-Forest is to take advantage of Local Sensitive Hashing and the generation of multiple, independent trees to create a consensus result. Our method is able to adapt to different decoy sets by utilizing decoy-specific energy functions to detect correct protein folds. Extensive tests on both Rosetta and I-TASSER decoy sets show that our method is up to 22 times faster than two recently published clustering/decoy selection methods Durandal and Calibur-lite. Our method also achieves better accuracy using both C_α_ RMSD and GDT-TS distance metrics for two different decoy sets.

While no clustering method or scoring function has yet been developed that can consistently identify the most correct structure among large decoy sets, we believe HS-Forest is a step in the right direction. We hope this idea can inspire the development of even better methods for correct fold detection, and that this concept of partial clustering may be seen to have applications to other scientific fields facing similar clustering challenges.

## Availability and requirements

**Project name**: HS-Forest

**Project homepage**: http://webdocs.cs.ualberta.ca/~jianjun/hsforest/

**Operating System**: Tested on Linux.

**Programming Language**: C++.

**Other requirements**: None.

**License**: GNU General Public License

## Competing interests

The authors declare that they have no competing interests.

## Authors’ contributions

All authors jointly developed the methods and wrote the article. They read and approved the final manuscript.

## Supplementary Material

Additional file 1 **Details of the Rosetta decoy sets.** The size of each decoy set is larger than 1000, and each decoy has the same length as the native structure in PDB. Click here for file

Additional file 2 Details of the I-TASSER decoy sets. Click here for file
