# Supplementary material for: An improved method to detect correct protein folds using partial clustering
Source: BMC Bioinformatics. 2013 Jan 16;14:11. doi: 10.1186/1471-2105-14-11 (PMC3626854; doi:10.1186/1471-2105-14-11)
Supplement: Additional file 1 — Details of the Rosetta decoy sets. The size of each decoy set is larger than 1000, and each decoy has the same length as the native structure in PDB. [file 1471-2105-14-11-S1.pdf]

Additional File #1: Details of the Rosetta decoy sets. The size of each decoy set is larger than 1000, and each decoy has the same length as the native structure in PDB.

Target protein	Number of decoys	Top Structure from Rosetta with HS-Forest		Speedup (Time of X/Time for HS-Forest)	
		Average Cα RMSD (Å) / TM-score	Standard Deviation Cα RMSD (Å) / TM-score	Durandal	Calibur-lite
1acf	3680	1.86 / 0.872	0.11 / 0.019	2.925	3.647
1bk2	15897	0.84 / 0.191	0.14 / 0.002	9.969	4.835
1bkr	7857	1.97 / 0.492	0.39 / 0.005	5.431	4.351
1bm8	64307	1.70 / 0.305	0.87 / 0.008	Ran out of memory (>16GB)	Ran out of time (>2h)
1cc8	11030	2.17 / 0.396	1.06 / 0.015	7.489	5.647
1cei	1778	1.22 / 0.273	0.09 / 0.003	1.791	3.805
1ctf	1135	1.33 / 0.080	0.05 / 0.001	1.418	2.957
1elw	3324	0.80 / 0.561	0.17 / 0.002	2.709	2.129
1enh	12965	2.12 / 0.263	0.03 / 0.003	8.677	6.690
1ew4	12660	1.56 / 0.878	0.08 / 0.015	8.618	6.562
1fna	7898	1.30 / 0.208	0.05 / 0.001	6.016	5.111
1iib	11798	1.92 / 0.324	0.60 / 0.003	8.863	5.684
1mjc	14845	2.48 / 0.387	0.05 / 0.005	10.92	6.139
1nps	9134	0.95 / 0.947	0.22 / 0.015	6.977	6.029
1opd	8332	1.15 / 0.910	0.09 / 0. 025	6.647	4.973
1poh	8274	2.83 / 0.701	0.17 / 0.060	6.380	4.643
1prq	17934	2.50 / 0.816	0.10 / 0.010	14.02	10.18
1r69	11073	1.23 / 0.889	0.30 / 0.023	7.803	6.263
1tig	1524	1.04 / 0.056	0.20 / 0.001	1.593	3.602
1ttz	12481	5.21 / 0.382	0.45 / 0.010	8.226	4.025
1tul	1727	1.10 / 0.165	0.50 / 0.002	1.722	3.118
1vcc	13624	3.40 / 0.665	0.08 / 0.006	8.889	8.882
1vkk	4775	2.43 / 0.249	0.05 / 0.001	3.407	3.730
1wdv	4492	1.65 / 0.379	0.51 / 0.008	3.489	3.344
2acy	6340	0.77 / 0.965	0.19 / 0.010	4.620	3.389
2chf	6184	1.53 / 0.545	0.27 / 0.007	4.493	4.044
2h28	6938	3.17 / 0.156	0.29 / 0.004	5.102	4.219
2he4	10758	3.15 / 0.845	0.35 / 0.010	7.106	4.229
2icp	8443	8.06 / 0.195	0.48 / 0.004	5.986	5.697
2vpt (t286)	3375	1.63 / 0.437	0.06 / 0.003	2.755	6.221
2h00 (t293)	3635	2.97 / 0.603	0.11 / 0.009	2.905	7.267
2hsj (t297)	3278	8.73 / 0.729	0.42 / 0.013	2.638	2.412
2hbo (t322)	8893	7.74 / 0.166	0.18 / 0.002	6.353	8.829
2i5t (t342)	1196	11.88 / 0.157	0.86 / 0.005	1.474	3.916
2hi6 (t357)	1587	6.07 / 0.454	0.38 / 0.012	1.695	5.162
