# Supplementary material for: An improved method to detect correct protein folds using partial clustering
Source: BMC Bioinformatics. 2013 Jan 16;14:11. doi: 10.1186/1471-2105-14-11 (PMC3626854; doi:10.1186/1471-2105-14-11)
Supplement: Additional file 2 — Details of the I-TASSER decoy sets. [file 1471-2105-14-11-S2.pdf]

Additional File #2: Details of the I-TASSER decoy sets.

Target protein	Number of decoys	Top Structure from GaFolder with HS-Forest	
		Average<br>Cα RMSD (Å) / TM-score	Standard deviation<br>Cα RMSD (Å) / TM-score
1af7__	12499	4.38 / 0.495	0.58 / 0.052
1ah9_	27498	3.12 / 0.643	0.15 / 0.013
1aoy_	32000	4.29 / 0.687	0.14 / 0.012
1b72A	12500	2.69 / 0.769	0.07 / 0.016
1cewI	19830	3.55 / 0.721	0.13 / 0.017
1cqkA	19999	1.66 / 0.860	0.05 / 0.008
1csp_	12500	2.38 / 0.720	0.05 / 0.008
1cy5A	32000	1.60 / 0.875	0.03 / 0.006
1di2A_	20000	2.37 / 0.720	0.10 / 0.029
1dtjA_	20000	1.95 / 0.789	0.11 / 0.016
1egxA	20000	2.57 / 0.774	0.07 / 0.007
1fadA	12500	3.63 / 0.589	0.06 / 0.009
1fo5A	20000	3.80 / 0.539	0.04 / 0.006
1g1cA	19997	2.66 / 0.772	0.09 / 0.010
1gpt_	32000	6.55 / 0.493	0.15 / 0.016
1gyvA	11508	3.43 / 0.765	0.07 / 0.013
1hbkA	20000	3.86 / 0.595	0.22 / 0.026
1jnuA	20000	2.62 / 0.750	0.04 / 0.002
1kviA	20000	2.26 / 0.684	0.06 / 0.016
1mkyA3	12500	5.17 / 0.417	0.12 / 0.007
1mla_2	12500	3.12 / 0.618	0.14 / 0.008
1n0uA4	12499	4.33 / 0.486	0.10 / 0.012
1ne3A	12500	7.02 / 0.386	0.61 / 0.038
1npsA	20000	2.21 / 0.776	0.11 / 0.017
1of9A	20000	3.62 / 0.537	0.03 / 0.007
1ogwA_	19998	1.25 / 0.863	0.10 / 0.016
1orgA	20000	2.61 / 0.758	0.09 / 0.014
1pgx_	20000	3.17 / 0.524	0.13 / 0.018
1r69_	20000	2.06 / 0.729	0.12 / 0.017
1shfA	20000	1.46 / 0.822	0.05 / 0.010
1sro_	20000	3.65 / 0.645	0.07 / 0.012
1ten_	20000	1.88 / 0.803	0.06 / 0.012
1tfi_	32000	4.92 / 0.514	0.20 / 0.021
1thx_	32000	2.26 / 0.831	0.05 / 0.003
1tig_	12500	3.97 / 0.557	0.26 / 0.036
256bA	20000	3.55 / 0.749	0.05 / 0.007
2a0b_	32000	2.44 / 0.809	0.05 / 0.010
2cr7A	12500	3.99 / 0.457	0.51 / 0.035
2f3nA	19999	1.95 / 0.711	0.16 / 0.026
2pcy_	20000	4.83 / 0.639	0.07 / 0.006
